# Manipulating nitrogen regulation in diazotrophic bacteria for agronomic benefit

**DOI:** 10.1042/BST20180342

**Published:** 2019-04-01

**Authors:** Marcelo Bueno Batista, Ray Dixon

**Affiliations:** Department of Molecular Microbiology, John Innes Centre, Colney Lane, Norwich NR4 7UH, U.K.

**Keywords:** ammonia excretion, nitrogen fixation, nitrogen regulation, PII signal transduction proteins

## Abstract

Biological nitrogen fixation (BNF) is controlled by intricate regulatory mechanisms to ensure that fixed nitrogen is readily assimilated into biomass and not released to the environment. Understanding the complex regulatory circuits that couple nitrogen fixation to ammonium assimilation is a prerequisite for engineering diazotrophic strains that can potentially supply fixed nitrogen to non-legume crops. In this review, we explore how the current knowledge of nitrogen metabolism and BNF regulation may allow strategies for genetic manipulation of diazotrophs for ammonia excretion and provide a contribution towards solving the nitrogen crisis.

## Introduction

Nitrogen is an essential element for all living organisms as it is part of the building blocks that constitute nucleic acids and proteins. Although gaseous nitrogen (N_2_) is abundant in the atmosphere, it cannot be assimilated by most living organisms. Only a selected group of Bacteria and Archaea, called diazotrophs, have evolved the ability to reduce N_2_ to generate NH_3_ in a process known as biological nitrogen fixation (BNF) catalysed by nitrogenase, an oxygen-sensitive enzyme complex. The reaction catalysed by the nitrogenase enzyme is an extremely energy-demanding process since reduction of 1 mol of N_2_ requires 16 mol of ATP *in vitro* [1–3]. However, the energetic penalty *in vivo* can increase up to 40 mol of ATP per mol of N_2_ in aerobic diazotrophs particularly at high oxygen tensions when respiratory activity has to be diverted to protect nitrogenase from oxygen damage [4,5]. Given this huge energy load, diazotrophs have evolved elegant strategies to tightly regulate the expression and activity of components required for BNF.

Fixed nitrogen is one of the most limiting nutrients for crop production. However, the application of chemical nitrogen fertilisers in agriculture has reached unsustainable levels that have resulted in increased greenhouse gas emissions [6,7] and other severe environmental consequences [8]. This has created a strong driver to provide a more sustainable alternative to synthetic nitrogen by engineering BNF in plants, either by engineering the legume symbiosis into cereals or by transferring the components required for nitrogenase activity into plant organelles such as chloroplast and mitochondria [9–12]. Significant advances towards the realisation of these two goals have been made [10], although both approaches are reliant on complex synthetic biology strategies, which will probably take considerable time to develop as viable products for farmers, due to the complexity of the bioengineering required and current restrictions on the use of genetically modified crops. A third approach could involve the enhancement of naturally occurring plant-associated diazotrophs [10,13–15] by generating strains that release fixed nitrogen to benefit the crop. Manipulation of soil diazotrophs can potentially provide a means to reduce the use of synthetic nitrogen fertilisers thus providing a short-term solution to the nitrogen crisis. In this review, we will focus on fundamental knowledge of the regulation of BNF in response to fixed nitrogen to explore how this process might be manipulated and decoupled from ammonium assimilation to generate enhanced diazotrophs that can more effectively deliver fixed nitrogen to benefit plant growth.

## Key players in nitrogen regulation of nitrogen fixation

Diazotrophic bacteria do not normally altruistically release ammonia to their environment since the processes of nitrogen fixation and nitrogen assimilation are intrinsically coupled and controlled by complex regulatory circuits that ensure that fixed nitrogen is readily assimilated into biomass rather than excreted from the cell. The components of these regulatory circuits are common to many Proteobacteria and include PII signal transduction proteins (e.g. GlnB and GlnK), additional enzymes that carry out reversible post-translational modification of proteins in response to metabolite control (GlnD and GlnE), and a two-component regulatory system (NtrB or NRII and NtrC or NRI) that controls the expression of many genes involved in nitrogen metabolism [16–18]. In diazotrophic Proteobacteria, these circuits interface with the master regulator of nitrogen fixation (*nif*) genes (NifA, a member of the bacterial enhancer-binding family [19]) to ensure that nitrogenase expression is stringently regulated by the demand for fixed nitrogen [20]. Gaseous ammonia that diffuses across the bacterial cell membrane under conditions of nitrogen fixation can be recovered by the high-affinity ammonium transporter (AmtB), although this invokes an energy-dependent futile cycle since ammonium uptake by AmtB is an electrogenic process [21,22].

**Figure 1.**
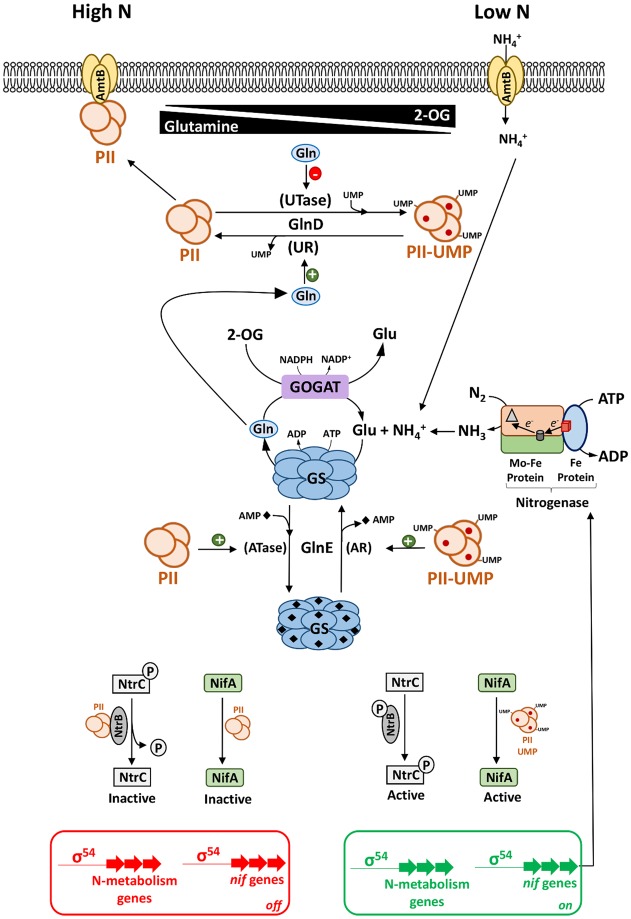
Regulation of nitrogen metabolism by PII signal transduction proteins. The term PII is generically used in this figure to represent various paralogs present in different organisms. Regulatory outputs triggered by ammonium excess (High N) and ammonium deficiency (Low N) are depicted on the left- and right-hand sides of the diagram, respectively. The bicyclic regulatory cascade involving GlnD (UTase/UR) and GlnE (ATase/AR), which controls GS activity and consequently the flux of nitrogen through the GS-GOGAT pathway, is shown in the centre of the figure. The carbon:nitrogen ratio, perceived by the concentrations of the regulatory metabolites 2-oxoglutarate (2-OG) and glutamine (Gln) controls the uridylylation status of the PII proteins and their interactions with diverse targets. These include control of NH_4_^+^ transport by AmtB (top), regulation of nitrogen metabolism by the two-component NtrB–NtrC system and regulation of nitrogen fixation by NifA (bottom). Under ammonium replete conditions (left), high levels of glutamine stimulate de-uridylylation of PII by the uridylyl-removing (UR) activity of GlnD. Non-modified PII interacts with AmtB to block NH_4_^+^ transport and stimulates the ATase activity of GlnE leading to GS adenylylation and impairment of NH_4_^+^ assimilation via the GS-GOGAT pathway. Non-modified PII also influences transcription of general nitrogen metabolism and nitrogen fixation (*nif*) genes by stimulating the phosphatase activity of NtrB, inactivating NtrC (via dephosphorylation) and finally by inhibiting directly or indirectly NifA activity. Under ammonium-limiting conditions (right), the cooperative binding of 2-OG (red circles) to PII trimers, combined with low levels of glutamine stimulate uridylylation of PII by the uridylyltransferase (UTase) activity of GlnD. Modified PII (PII-UMP) is no longer able to interact with AmtB, allowing NH_4_^+^ uptake. In addition, PII-UMP stimulates the AR activity of GlnE leading to GS deadenylylation and activation of NH_4_^+^ assimilation via the GS-GOGAT pathway. Finally, PII-UMP is no longer able to stimulate NtrB phosphatase activity, leading to NtrC phosphorylation and activation of nitrogen metabolism genes. PII-UMP also signals low-nitrogen levels to NifA, triggering its activation and transcription of *nif* genes, encoding structural and accessory genes required for nitrogenase activity (bottom right). For the sake of simplicity, the binding of ATP or ADP to PII is not shown in this figure. The nitrogenase enzyme components, MoFe protein and Fe protein, are shown as a rounded rectangle and oval, respectively (centre–right). The Fe-S cluster in the Fe protein is indicated by the red cube and the P and FeMoco clusters in the MoFe protein are shown as a cylinder and triangle, respectively.

In Proteobacteria, the nitrogen status is primarily sensed by trimeric PII signal transduction proteins, such as GlnB and GlnK, which can integrate various metabolic signals including the levels of glutamine, ADP, ATP, and 2-oxoglutarate to control both nitrogen fixation and nitrogen assimilation (for reviews, see [17,18,23–25]). The competitive binding of ATP and ADP to PII confers modulation of its activity in response to the energy status. In addition, the binding of 2-oxoglutarate provides a readout of the nitrogen and carbon status to regulate PII activity [26] (for reviews, see [23,24,27]). 2-oxoglutarate is an important signalling metabolite in this context since it lies at the interface of carbon and nitrogen metabolism, providing carbon skeletons for nitrogen assimilation from the TCA cycle [27]. In Proteobacteria, PII proteins are subject to post-translational modification by the bifunctional enzyme GlnD (uridylyltransferase/uridylyl-removing enzyme — UTase/UR), which can add uridylyl groups to PII or remove uridylyl groups from PII-UMP, depending on the nitrogen status, thus modulating how PII proteins interact with their targets [28,29]. Under ammonium-sufficient (High N) conditions, high levels of glutamine, a metabolic signal of the nitrogen status, result in the stimulation of the uridylyl-removing activity of GlnD, consequently resulting in the de-uridylylation of PII (Figure 1). The non-uridylylated form of the PII protein GlnK interacts with the ammonium transporter AmtB to inhibit ammonium uptake [30,31], whereas non-modified GlnB interacts with NtrB to stimulate dephosphorylation of NtrC [32,33] (Figure 1), switching off transcription of various genes related to nitrogen metabolism (for reviews, see [17,34]). Under ammonium-limiting conditions (Low N), when glutamine levels are low, GlnD uridylylates PII proteins modifying their output target responses. Fully uridylylated PII (PII-UMP3) can no longer interact with AmtB and NtrB, thus enabling ammonium uptake and stimulating NtrC phosphorylation, respectively (Figure 1). Phosphorylated NtrC (NtrC-P) then activates the expression of σ^54^-dependent genes important for nitrogen metabolism [17,34]. The alternative σ^54^ factor controls not only genes related to nitrogen metabolism but also other physiological traits such as motility, virulence, and biofilm formation [35]. In Proteobacteria, the number of PII homologues can vary, for example, from one in *Azotobacter vinelandii*, two in *Escherichia coli* and *Klebsiella oxytoca* M5a1 (also known as *K. pneumoniae* M5a1 [36] or *K.* sp. M5a1 [37]) to three in *Azoarcus olearius* BH72 (formerly known as *A.* sp. BH72, see [38]) and *Rhodospirillum rubrum* [24]. The presence of multiple PII homologues may enable hierarchical regulatory cascades where each PII homologue interacts with different targets to fulfil distinct roles. For example, in *K. oxytoca* M5a1 GlnB and GlnK perform relatively discrete functions in which GlnK is required to control ammonium uptake via interaction with AmtB, whereas GlnB regulates the phosphorylation state of NtrC via interaction with NtrB [39,40]. In addition, PII homologues perform discrete roles in the signalling cascade that control nitrogenase switch-off in *Azospirillum brasilense* and *R. rubrum* as discussed below.

Nitrogen assimilation primarily occurs via the synthesis of glutamate from ammonium and 2-oxoglutarate and is catalysed by two alternative pathways, involving either the enzyme glutamate dehydrogenase (GDH) or the GS-GOGAT pathway which combines the activities of the enzyme glutamine synthetase (GS) and glutamate synthase (GOGAT) (see reference [17] for a comprehensive review). GDH catalyses the reductive amination of 2-oxoglutarate to glutamate at the expense of 1 NADPH. As the enzyme does not require ATP and has a relatively high *K*_m_ (∼1 mM) for ammonium [41], it operates during growth in high-ammonium and low-carbon/energy conditions. In contrast, the GS-GOGAT pathway, which is ubiquitous in Bacteria, operates in relatively low-ammonium and high-carbon/energy conditions since GS has at least a 10-fold lower *K*_m_ for ammonium [42] compared with GDH and requires ATP to catalyse the amidation of glutamate to glutamine. The reaction catalysed by GOGAT results in the subsequent transfer of the amide group from glutamine to 2-oxoglutarate in a reductive reaction that requires NADPH and generates two molecules of glutamate (Figure 1).

In *E. coli* and in most Proteobacteria, ammonia assimilation is fine-tuned according to the nitrogen status of the cell, via modulation of GS activity by GlnE, a bifunctional adenylyltransferase/adenylyl-removing (ATase/AR) enzyme [43]. Adenylylation of GS reduces its activity and the reversible reactions carried out by GlnE are regulated by the intracellular level of glutamine and the uridylylation status of the PII proteins. Under nitrogen-sufficient conditions, GS adenylylation is stimulated by unmodified PII and by high levels of glutamine, whereas the deadenylylation reaction is stimulated by modified PII and by 2-oxoglutarate under low-nitrogen levels. Therefore, the uridylylation state of PII proteins and their binding to 2-oxoglutarate, influences GlnE activity, and hence the activity of GS (Figure 1). The integration of metabolic signals by two nucleotidyltransferases, GlnD (UTase/UR) and GlnE (ATase/AR), thus forms a bicyclic-cascade to control GS activity (reviewed in [17]).

The PII proteins also play a pivotal role in coupling nitrogen fixation with ammonium assimilation by signalling the nitrogen status to the nitrogen fixation machinery at both the transcriptional and post-translational levels. In many diazotrophic Proteobacteria, expression of the σ^54^-dependent activator NifA is controlled by the two-component NtrB–NtrC nitrogen regulatory system in response to covalent modification and the ligand-binding state of the PII proteins [18,44–46] (Figure 1). In addition, the activity of NifA itself is stringently regulated either directly or indirectly by PII signal transduction proteins. In many cases, particularly outside the Gammaproteobacteria, NifA seems to be intrinsically oxygen-sensitive and the nitrogen signal transduction mechanism involves direct interaction with PII proteins [20]. In *A. brasilense*, *Herbaspirillum seropedicae* and *R. rubrum*, uridylylated PII proteins are required to activate NifA when nitrogen is limiting, whereas in *Rhodobacter capsulatus*, *Azorhizobium caulinodans* and *Gluconacetobacter diazotrophicus*, the non-modified form of PII seems to be required to inhibit NifA activity when nitrogen is abundant [24,47]. In another group of diazotrophs initially identified in representatives of Gamma (*K. oxytoca*, *A. vinelandii* and *Pseudomonas stutzeri* A1501) and Beta (*A. olearius*) proteobacteria, NifA activity is controlled by a partner protein NifL, which senses environmental cues and interacts with PII proteins, to modulate NifA activity in response to the nitrogen status. In *A. vinelandii*, the non-modified form of GlnK is required for the formation of the inhibitory complex between NifL and NifA under nitrogen excess conditions [48,49], as opposed to *K. oxytoca*, where GlnK (irrespective of its modification state) is required to relieve inhibition of NifA activity by NifL under nitrogen-limiting conditions [39,50].

**Figure 2.**
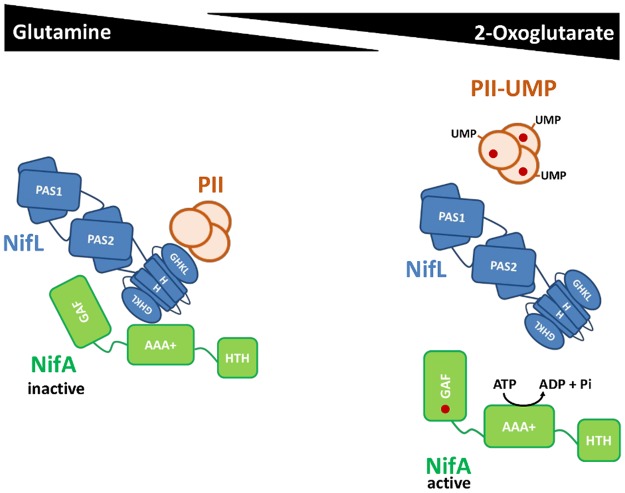
Nitrogen regulation of NifA activity by NifL and GlnK in *A. vinelandii*. Under nitrogen excess conditions, high levels of glutamine stimulate de-uridylylation of PII, resulting in the formation of a ternary PII–NifL–NifA complex that inhibits NifA activity (left-hand side). Upon a shift to low nitrogen, where glutamine levels are low and 2-OG levels (red circles) are high, the UTase activity of GlnD is stimulated, resulting in uridylylation of PII saturated with 2-OG (red circles). Under these conditions, PII-UMP is no longer able to interact with NifL. The complex between NifL and NifA dissociates to release NifA activity, provided that the regulatory GAF domain of NifA is saturated with 2-OG (red circle, right-hand side). Under conditions of carbon limitation, NifL can still form a complex with NifA and inhibit its activity, since binding of 2-OG to the GAF domain of NifA is required to bypass inhibition by NifL, even if PII is uridylylated.

The *A. vinelandii* NifL–NifA system has been extensively characterised at both the genetic and biochemical levels (for details, see reviews [48,49]). NifL senses the redox status via a FAD cofactor in its N-terminal PAS domain and interacts with GlnK via a C-terminal nucleotide-binding domain to respond to the nitrogen status. Under conditions that result in oxidation of the FAD cofactor (excess oxygen), NifL forms a binary complex with NifA that inhibits its activity, irrespective of the nitrogen status. Upon a shift to reducing conditions (low oxygen), NifL controls NifA activity according to the nitrogen status as signalled by the PII protein, GlnK. Under nitrogen excess conditions when GlnK is present in the non-uridylylated form, a ternary GlnK–NifL–NifA complex is formed in which NifA is inactive (Figure 2, left). Under nitrogen-limiting conditions, uridylylation of GlnK prevents the interaction with NifL leading to dissociation of the ternary complex (Figure 2, right) and consequent activation of NifA. However, activation of NifA under low-nitrogen conditions is also dependent on the carbon status, since binding of 2-OG to the regulatory GAF domain of NifA is required to prevent the formation of the inhibitory NifL–NifA binary complex [51–53]. Therefore, in addition to the response of NifL to the oxygen and nitrogen status, the interaction of NifL with NifA is modulated by carbon availability, since the ability of NifA to escape from inhibition by NifL is dependent on the binding of 2-oxoglurate to the GAF domain.

## Post-translational control of nitrogenase activity

**Figure 3.**
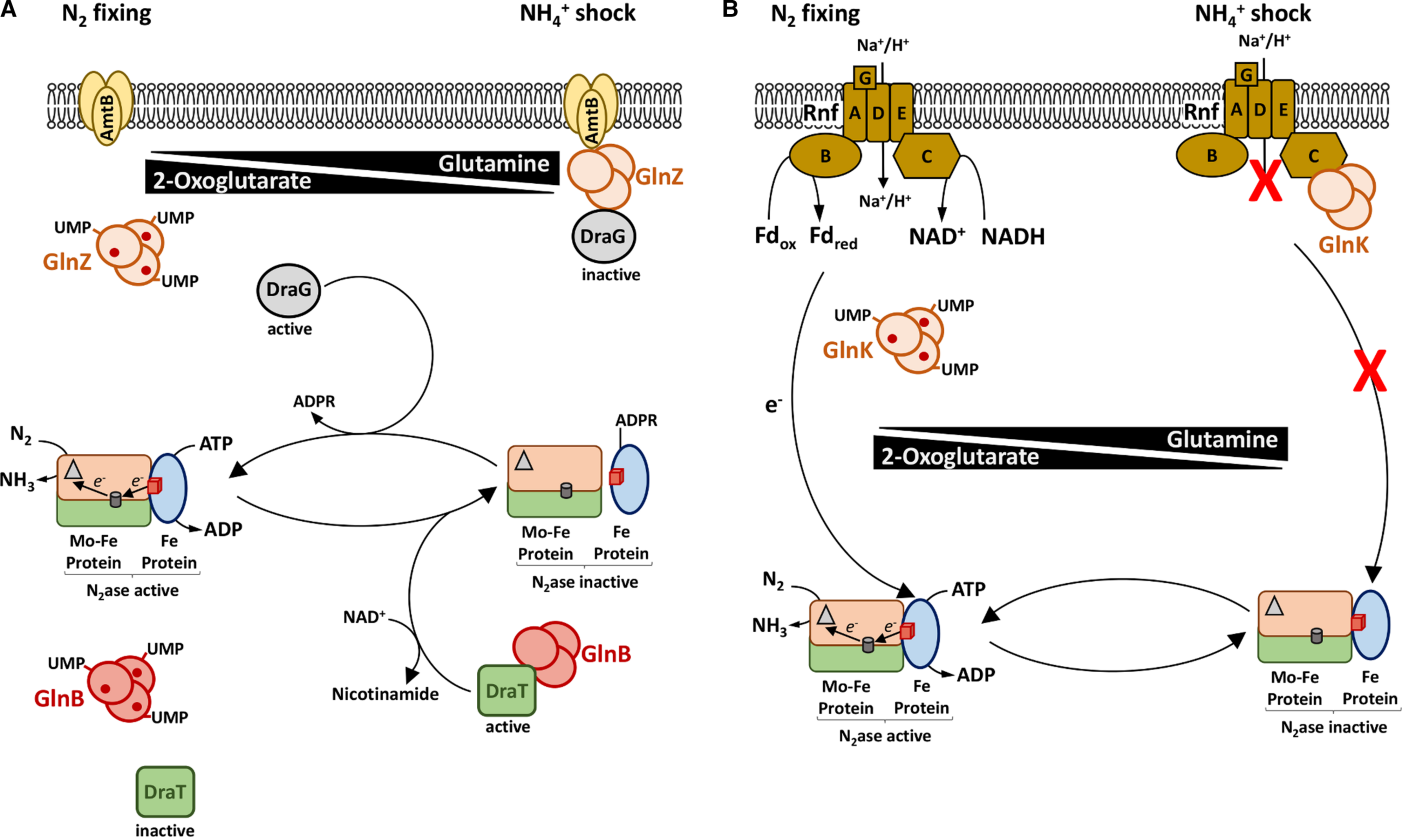
Nitrogenase switch-off mechanisms in response to NH_4_^+^ shock. (**A**) Post-translational modification of nitrogenase by ADP ribosylation, depending upon the PII proteins, DraT and DraG in *A. brasilense*. Under nitrogen-fixing conditions, GlnB and GlnZ are fully uridylylated and saturated with 2-OG (red circles) and therefore cannot interact with DraT (ADP-ribosyltransferase) or DraG (ADP-ribosyl glycohydrolase). Under such conditions, DraT is inactive, whereas DraG is located in the cytosol and active. Consequently, nitrogenase Fe protein is not ADP-ribosylated, and nitrogenase is active. After a shift to high NH_4_^+^, increased glutamine levels stimulate de-uridylylation of GlnB and GlnZ. Unmodified GlnZ becomes membrane-bound via its interaction with AmtB and also sequesters DraG to the membrane, forming an AmtB–GlnZ–DraG ternary complex, that sterically occludes interaction with nitrogenase Fe protein. In addition, unmodified GlnB is then able to interact with DraT, stimulating its activity, leading to ADP ribosylation of the Fe protein, which inhibits nitrogenase activity. (**B**) Physiological switch-off of nitrogenase activity dependent upon GlnK and the Rnf1 complex in *A. olearius* BH72. Under N_2_-fixing conditions, an electrochemical gradient across the membrane is used to drive the Rnf1 complex to reduce FdxN (Fd_red_), which serves as the electron donor to support nitrogenase activity. Upon a shift to high NH_4_^+^ conditions, increased glutamine levels stimulate de-uridylylation of GlnK, which then interacts with the RnfC subunit of the Rnf1 complex to block its activity, thus preventing electron flow to nitrogenase. Nitrogenase component proteins are illustrated as in Figure 1.

In addition to the complex signal transduction cascades that couple nitrogen fixation to ammonium assimilation, the activity of the nitrogenase enzyme itself is regulated by fixed nitrogen availability. At least three different mechanisms, involving PII proteins, are known to switch-off nitrogenase activity under excess nitrogen conditions in Proteobacteria. The first mechanism involves inactivation of nitrogenase Fe protein by ADP ribosylation, catalysed by the DraT (ADP-ribosyltransferase) enzyme and has been extensively characterised in *R. rubrum* and *A. brasilense* (for a detailed review, see [54]). This post-translational covalent modification can be reversed by another enzyme with opposing activity named DraG (ADP-ribosyl glycohydrolase). Interestingly, the activity of both enzymes is regulated by interaction with PII proteins depending on the nitrogen status. Under excess nitrogen conditions, non-modified PII (GlnB) interacts with DraT-stimulating ADP ribosylation [55–57]. In addition, a second PII paralog, GlnZ in *A. brasilense* (GlnK in *R. rubrum*), mediates formation of a ternary complex between AmtB, GlnZ and DraG, that sequesters DraG to the membrane, inhibiting its ability to remove the covalent modification from nitrogenase [54,58,59]. Under conditions of nitrogen limitation, the uridylylated forms of PII cannot interact with either DraT or DraG. Under these circumstances, DraT is inactive and DraG is active, leading to the removal of the ribosyl modification from the nitrogenase Fe protein, thus reactivating nitrogenase (Figure 3A). A second mechanism for nitrogenase switch-off apparently involves inhibition of the Rnf1 electron transport system (encoded by *rnfABCDE*), which potentially provides a dedicated electron transfer pathway to nitrogenase in *A. vinelandii* [60]. Genes encoding the Rnf1 complex are co-located with the *nifLA* operon in diverse diazotrophs (see below). The interaction between the PII signal transduction GlnK and the Rnf1 complex has been proposed to control electron transfer to nitrogenase in *A. olearius* BH72. Under conditions of ammonium shock, the non-modified form of GlnK interacts with the RnfC component of the membrane-bound Rnf1 complex. This is likely to decrease electron transport and potentially disrupt the electron flow to nitrogenase [61] (Figure 3B). A third unknown mechanism, yet to be described in detail, is responsible for nitrogenase switch-off in organisms lacking both DraT–DraG and the Rnf electron transport system. In *H. seropedicae*, for example, rapid nitrogenase switch-off during ammonium upshift requires both the *amtB* and *glnK* genes, suggesting that this elusive mechanism may require formation of an AmtB-GlnK complex in the membrane [24,54].

## New insights from NifL–NifA distribution in Proteobacteria

**Figure 4.**
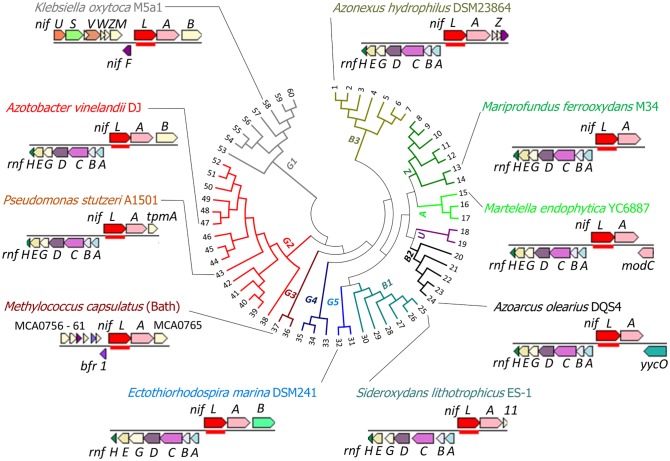
The NifL–NifA regulatory system is widespread in Proteobacteria. Phylogenetic analysis of NifL from selected Proteobacteria is shown in the centre of the figure. The tree was constructed using the maximum-likelihood method based on the Le_Gascuel_2008 model [81]. The tree with the highest log-likelihood (−8971.99) is shown and is drawn to scale, with branch lengths measured by the number of substitutions per site. The analysis involved 60 amino acid sequences. All positions containing gaps and missing data were eliminated with a total of 151 positions in the final dataset. Evolutionary analyses were conducted using MEGA7 [82]. The genomic neighbourhood diagrams of selected bacterial species within phylogenetic groups are also indicated and were generated using IMG web resources [83]. The different phylogenetic groups are colour coded and named as follows: Gammaproteobacteria, G1, G2, G3, G4 and G5; Betaproteobacteria, B1, B2 and B3; Alphaproteobacteria, A; Zetaproteobacteria, Z and unclassified, U. NCBI sequence IDs for the proteins used in the phylogenetic tree are as follows: 1: WP_083651365; 2: WP_049758561; 3: AQR63681; 4: WP_026687500; 5: OHC64658; 6: OHC71058; 7: WP_091932006; 8: OIQ03030; 9: PIO90509; 10: PJC68217; 11: OIO71574; 12: PIV30920; 13: WP_018295154; 14: PIW46261; 15: WP_045682635; 16: WP_069958434; 17: SBW07101; 18: PJA24783; 19: OIO54772; 20: WP_091193542; 21: WP_107492198; 22: WP_108949303; 23: WP_083831893; 24: WP_084018173; 25: WP_013028993; 26: OHC81074; 27: OGS92673; 28: OYY92669; 29: SFW97644; 30: WP_079433087; 31: OUD15751; 32: WP_090253894; 33: OTE98078; 34: OAI29411; 35: OQW74710; 36: WP_051332068; 37: WP_017365054; 38: WP_104002745; 39: WP_085156478; 40: WP_045825321; 41: WP_075185744; 42: WP_096085066; 43: WP_014596155; 44: WP_102654323; 45: WP_090444344; 46: WP_065835961; 47: WP_012703539; 48: WP_090621470; 49: WP_089169635; 50: WP_090349205; 51: WP_092432206; 52: WP_090313089; 53: WP_088237514; 54: WP_096834788; 55: WP_070612308; 56: WP_087833496; 57: WP_100685014; 58: WP_082236990; 59: WP_049089492; 60: WP_098362797.

Although the NifL–NifA system was originally considered to be restricted to diazotrophic Gammaproteobacteria and a single representative of the Betaproteobacteria [20], the increasing availability of sequenced genomes has revealed that this system is widespread amongst Proteobacteria, including representatives from the Gamma, Alpha, Beta and Zeta subgroups. Our phylogenetic analysis of NifL proteins, combined with genome neighbourhood analysis, reveals that in most groups, the *nifLA* operon is closely associated with an *rnf* operon that is apparently divergently transcribed (Figure 4). Notably in *A. vinelandii* DJ [62], *P. stutzeri* A1501 [63] and *A. olearius* BH72 [64], the *rnf1* operon is subject to regulation by NifA. As mentioned above, these linked *rnf* genes are likely to provide a dedicated electron transfer pathway to nitrogenase [60]. Whether the correlation of phylogenetic clades with genomic neighbourhood is linked with differences in signal transduction mechanism and/or physiological lifestyles has yet to be determined, but intriguingly we found only two clades (G1 and G3) where the association between the *rnf* and *nifLA* operons is not observed (Figure 4). Organisms in the G1 clade, comprising various representatives of the Enterobacteriaceae, are facultative anaerobes that fix nitrogen under anaerobic conditions. As mentioned above, there are fundamental differences in signal transduction mechanisms involving NifL–NifA and GlnK in *K. oxytoca* (G1 clade) compared with *A. vinelandii* (G2 clade), which might reflect physiological lifestyles and a requirement for the Rnf1 complex to support electron flux to nitrogenase activity under aerobic conditions. The absence of co-location between *rnf* and *nifLA* in group G3, represented by the methanotrophs (*Methylococcus capsulatus* and *Methylocaldum szegediense*), is more difficult to rationalise, given that these organisms can fix nitrogen under microaerobic conditions [65]. Overall, the widespread co-location of *rnf1* and *nifLA* operons in diazotrophic genomes may reflect physiological traits within particular phylogenetic groups and can possibly shed light into signal transduction mechanisms controlling nitrogen fixation in these organisms. Although it is difficult to speculate at this stage, one potential hypothesis for the neighbourhood co-location of *rnf1* with *nifLA*, is that it provides integration of the regulation of *nif* gene transcription with the expression of electron transfer components required for nitrogenase activity, in addition to the possibility of conferring physiological switch-off of nitrogenase in response to ammonia.

## Engineering ammonia excretion in diazotrophs

The complex regulatory circuits described above ensure that components required for the biosynthesis and activity of nitrogenase are expressed only under demanding physiological conditions. They also ensure that regulation of nitrogen fixation and ammonia assimilation are intertwined, so that fixed nitrogen becomes readily available to support bacterial growth instead of being altruistic released to the environment. Knowledge of the control hierarchies and feedback regulation in response to the nitrogen status provide the potential for metabolic engineering of plant-associated diazotrophs to excrete surplus ammonium to benefit plant growth. The obvious targets for this metabolic engineering approach include perturbation of (a) ammonium assimilation (e.g. by introducing mutations that influence GS or GOGAT activity or that modulate fine-tuning of GS activity by post-translational modification), (b) *nif* gene expression (e.g. by engineering constitutive transcriptional activation by the master regulator NifA), (c) nitrogenase switch-off mechanisms (e.g. by perturbing activity of the DraT–DraG enzymes or the PII signal transduction components that modulate their activity), or (d) coupling between the PII uridylylation and GS adenylation states in response to nitrogen (e.g. by altering uridylyl and adenylyl removal activities of GlnD and GlnE, respectively). Ideally, these metabolic perturbations should result in the excretion of a major proportion of the ammonia produced by nitrogenase while maintaining assimilation rates at sufficient levels to support bacterial fitness.

To date, there are several examples in the literature where one or more of the above-mentioned strategies have been utilised to generate ammonia-excreting strains. For example, deletion of *nifL* in *A. vinelandii*, which removes all regulatory inputs controlling NifA activity, results in the excretion of millimolar levels of ammonia [66–69]. However, these mutations are apparently unstable, potentially due to the energetic penalty associated with constitutive synthesis of high levels of nitrogenase. The release of ammonia by these strains may imply that the surfeit of ammonia produced by nitrogenase results in the assimilation of excess glutamine, resulting in feedback regulation of GS. Introduction of *glnA* substitution that decreases GS activity results in increased ammonium release as anticipated [70]. In some cases, engineering strains with deficiencies in GS alone have provided useful strategies for ammonia release. In *A. brasilense*, mutant strains selected for resistance to ethylenediamine (EDA) have been shown to excrete ammonia [71,72]. In these strains, the expression and/or activity of GS appear to be affected, suggesting that ammonium assimilation is compromised. In one of these EDA-resistant mutants (strain HM053), ADP ribosylation of nitrogenase upon ammonium shock is also impaired [73]. Therefore, in *A. brasilense* strain HM053, the combination of low GS activity (which is not related to increased GS adenylylation [73]) with impaired nitrogenase switch-off apparently enables ammonia excretion. Disruption of PII signal transduction pathways has also been utilised to release ammonia in *A. caulinodans*, where the GlnB and GlnK proteins are required for both deadenylylation of GS and inhibition of NifA activity in response to nitrogen levels. A mutant strain with insertions in both of these genes resulted in constitutive NifA activity, decreased GS activity and consequently the ability to excrete ammonia, albeit at micromolar levels [74].

In some cases, ammonia-excreting strains have provided evidence that BNF can contribute to substantial plant growth promotion in model experiments in which single bacterial strains are used to inoculate plant varieties grown on sterile substrates. For example, inoculation with ammonium-excreting strains of *A. brasilense* provides substantial growth benefit to wheat plants [72,73] and inoculation of *Setaria viridis* with *A. brasilense* strain HM053 has been demonstrated to provide sufficient nitrogen to support plant growth [75]. Mis-regulation of the *P. stutzeri* A1501 *nif* gene cluster when engineered into *Pseudomonas protegens* Pf-5 leads to ammonia excretion and plant growth promotion [76]. In some cases, the beneficial effects of ammonium-excreting strains on plant growth extend to non-sterile conditions in the greenhouse [77–79]. However, unequivocal evidence to convincingly demonstrate that such strains can robustly contribute to the N nutrition of the crop under field conditions is currently lacking. Since diazotrophic strains expressing nitrogenase constitutively are likely to encounter severe fitness penalties in competitive soil environments, it would be preferable to engineer rhizosphere-dependent switches that only trigger ammonium excretion when the bacteria are either closely associated or inside plant roots. Therefore, in addition to engineering the bacteria, manipulation of the plant partner may be required to ensure appropriate signalling and nutrient exchange to create an efficient synthetic symbiosis between the plant and the microbe [14,15].

**Perspectives**

The use of BNF to tackle the nitrogen problem in agriculture has been attracting research efforts from many laboratories across the globe. The two major synthetic biology approaches to this problem: (a) engineering the nodule symbiosis in non-legumes [9,80] and (b) engineering expression of components required for nitrogenase biosynthesis and activity in plant organelles [11,12], may, in the long term, enable crops to fix their own nitrogen and have brought optimism to the field. However, the development of robust systems may take several decades to deliver to farmers, due to the complex nature of the engineering required and the regulatory issues surrounding the use of GM crops. In the meantime, the strategies discussed here for improving naturally occurring diazotrophs without using extensive genetic manipulation may provide an alternative to reduce the environmental burden caused by excessive use of chemical nitrogen fertilisers. Although most of the current manipulations employed to engineer ammonium-excreting diazotrophs have been somewhat brute force (e.g. by introducing foreign promoters or selecting mutations that have not been characterised at the mechanistic level), we believe that a thorough understanding of nitrogen economy in plant-associated diazotrophic bacteria and its relationship with the carbon status will enable us to introduce subtle genome modifications that result in conditional release of ammonia, for example, in response to carbon availability in plant root exudates. Since these genetic manipulations can be achieved without the introduction of foreign DNA, such strategies may lead to the development of novel diazotrophic bioinoculants, compatible with current biosafety regulations.

## Abbreviations

ATase/AR: adenylyltransferase/adenylyl-removing
BNF: biological nitrogen fixation
EDA: ethylenediamine
GDH: glutamate dehydrogenase
GOGAT: glutamate synthase
GS: glutamine synthetase
UTase/UR: uridylyltransferase/uridylyl-removing
